# Nine-year seroepidemiological study of severe fever with thrombocytopenia syndrome virus infection in feral horses in Cape Toi, Japan

**DOI:** 10.1186/s12917-024-04042-7

**Published:** 2024-05-11

**Authors:** Hirohisa Mekata, Kentaro Yamada, Kazumi Umeki, Mari Yamamoto, Akihiro Ochi, Kunihiko Umekita, Ikuo Kobayashi, Takuya Hirai, Tamaki Okabayashi

**Affiliations:** 1 Division of Research & Inspection for Infectious Diseases, Center for Animal Disease Control, University of Miyazaki, 1-1 Gakuen-kibanadai Nishi, Miyazaki, 889-2192 Japan; 2 Department of Veterinary Science, Faculty of Agriculture, University of Miyazaki, 1-1 Gakuen-kibanadai Nishi, Miyazaki, 889-2192 Japan; 3 Department of Microbiology, Faculty of Medicine, Oita University, 1-1 Idaigaoka, Hasama-machi, Yufu City, Oita 879-5593 Japan; 4 Division of Respirology Rheumatology, Infectious Diseases and Neurology, Internal Medicine, Department of Internal Medicine, Faculty of Medicine, University of Miyazaki, 5200-Kihara, Kiyotake, Miyazaki 889-1692 Japan; 5 Equine Research Institute, Racing Association, 1400-4 Shiba, Shimotsuke, Tochigi 329-0412 Japan; 6 Field Science Center, Faculty of Agriculture, University of Miyazaki, 10100-1 Shimanouchi, Miyazaki, 880-0121 Japan

**Keywords:** Horses, Japan, SFTS Phlebovirus, Ticks, Wild animals, Zoonoses

## Abstract

Severe fever with thrombocytopenia syndrome (SFTS) is a fatal zoonosis caused by ticks in East Asia. As SFTS virus (SFTSV) is maintained between wildlife and ticks, seroepidemiological studies in wildlife are important to understand the behavior of SFTSV in the environment. Miyazaki Prefecture, Japan, is an SFTS-endemic area, and approximately 100 feral horses, called Misaki horses (*Equus caballus*), inhabit Cape Toi in Miyazaki Prefecture. While these animals are managed in a wild-like manner, their ages are ascertainable due to individual identification. In the present study, we conducted a seroepidemiological survey of SFTSV in Misaki horses between 2015 and 2023. This study aimed to understand SFTSV infection in horses and its transmission to wildlife. A total of 707 samples from 180 feral horses were used to determine the seroprevalence of SFTSV using enzyme-linked immunosorbent assay (ELISA). Neutralization testing was performed on 118 samples. In addition, SFTS viral RNA was detected in ticks from Cape Toi and feral horses. The overall seroprevalence between 2015 and 2023 was 78.5% (555/707). The lowest seroprevalence was 55% (44/80) in 2016 and the highest was 92% (76/83) in 2018. Seroprevalence was significantly affected by age, with 11% (8/71) in those less than one year of age and 96.7% (435/450) in those four years of age and older (*p* < 0.0001). The concordance between ELISA and neutralization test results was 88.9% (105/118). SFTS viral RNA was not detected in ticks (*n* = 516) or feral horses. This study demonstrated that horses can be infected with SFTSV and that age is a significant factor in seroprevalence in wildlife. This study provides insights into SFTSV infection not only in horses but also in wildlife in SFTS-endemic areas.

## Background

Severe fever with thrombocytopenia syndrome (SFTS) is an emerging tick-borne zoonotic disease in East Asia that is fatal in 10–27% of patients [1–3]. The causative agent of SFTS is formally named *Bandavirus dabieense*, which belongs to the family Phenuiviridae and the genus *Bandavirus* (International Committee on Taxonomy of Viruses, March 2023; MSL #38). Because the formal name has changed frequently, this study used the most common name, SFTS virus (SFTSV). SFTSV was first detected in humans with acute febrile illnesses in China in 2009 [4]. Subsequently, SFTSV endemics have been confirmed not only in China but also in South Korea and Japan [5–7]. SFTSV has also been confirmed in other Asian countries, such as Vietnam, Pakistan, Thailand, and Myanmar [8–11]. However, detailed information on SFTS, including the number of people in these countries who are infected and die from SFTSV, is lacking.

SFTSV can infect a wide range of animals through exposure to ticks. Controlling SFTSV transmission in accessible animals is a critical component of public health because sick animals are a direct source of SFTSV infection [12–14]. Domestic cats are more likely to develop severe symptoms of SFTSV infection than other animals [15]. Although symptoms are severe in domestic dogs [16], experimentally infected dogs have shown little to no symptoms [17]. Therefore, it is believed that domestic dogs are more resistant to SFTSV than cats, and only a proportion of infected dogs develop severe symptoms. Anti-SFTSV antibodies and SFTS viral RNA have been detected in many livestock, wild rodents, and wild mammals [18–23]. However, the clinical signs of SFTSV infection in these animals remain unclear. The only exception is the feline cheetah, which has been reported to exhibit severe symptoms when infected with SFTSV [24]. SFTSV infection in horses is no exception, and few studies have examined [25, 26].

As SFTSV is considered to establish an interspecific cycle of infection between wildlife and ticks in Japan [19, 22, 23], the rate of SFTSV infection in wildlife could be correlated with the rate of viral possession in ticks. Therefore, monitoring SFTSV infections in wildlife is important for predicting SFTS outbreaks in humans. Miyazaki Prefecture, located in southeastern Kyushu, has the highest number of recorded cases of SFTS per capita in Japan [27]. In this study, we conducted a seroepidemiological survey of SFTSV in feral horses living in Cape Toi, Miyazaki Prefecture, Japan, between 2015 and 2023. Feral horses are an indigenous Japanese breed called the Misaki horse (*Equus caballus*), and approximately 100 of these horses live in the wild in Cape Toi. Their breeding areas have been designated as natural monuments in Japan. Although they are managed in a wild-like manner, each horse is uniquely identified to determine its age and the time of seroconversion to SFTSV. This study will be useful for understanding the transmission cycle of SFTSV in wildlife as well as SFTSV in horses.

## Methods

### Samples

Misaki horses were captured every September by the Cape Toi Management Association and the Faculty of Agriculture, University of Miyazaki, for worming and blood sampling. Blood samples were collected for the purpose of quarantine. A total of 707 residual serum samples from 180 feral horses collected between 2015 and 2023 were analyzed in this study.

A total of 293 residual serum samples from active Thoroughbred racehorses (*Equus caballus*) were obtained from the Equine Research Institute, Japan Racing Association. The blood were collected for the purpose of quarantine. No individual information associated with the sample, such as name, age, sex, or place of production was provided.

A total of 516 ticks were collected from Cape Toi between November 2021 and November 2022. Sampling was conducted six times in total at two-month intervals, with a maximum of 104 ticks collected per sampling. Adult and nymphal ticks were collected from the vegetation by dragging flannel sheets. The detail tick collection and species identification methods have been described in our previous studies [28].

### Enzyme-linked immunosorbent assay (ELISA)

A double-antigen ELISA was used to detect anti-SFTSV nucleocapsid protein (NP) antibodies [29]. The serum sample was heat-treated at 56°C for 30 min, centrifuged at 800 × *g* for 3 min, and the supernatant was diluted 25-fold with 20-fold diluted Blocking One (Nacalai tesque, Kyoto, Japan) before use in the ELISA. The details of the recombinant SFTSV NP (rSFTSV-NP), rSFTSV-NP labeled with horseradish peroxidase (HRP), rabbit serum immunized with rSFTSV-NP used as a positive control, and the standard purified rabbit anti-SFTSV NP IgG used to construct the calibration curve are described in our previous report [29]. One hundred microliters of rSFTSV-NP diluted to 3 µg/mL in phosphate-buffered saline (PBS) with bovine serum albumin was added to half of the wells of a 96-well microtiter plate (F96 Maxisorp, Thermo Fisher Scientific, Waltham, MA, USA), and 100 µL of PBS with bovine serum albumin was added to the other half and coated overnight at 4°C. After washing with Tris-buffered saline with Tween 20 (TBS-T) and blocking with 5-fold diluted Blocking One for 1 h at 20–26°C, 100 µL of the diluted serum samples or rabbit anti-SFTSV standard IgG was added to both coated and uncoated wells and incubated for 2 h at 20–26°C with shaking. After washing with TBS-T, 100 µL of HRP-conjugated rSFTSV-NP diluted 2000-fold with 20-fold diluted Blocking One was added and incubated for 1 h at 20–26°C with shaking and light protection. After washing with TBS-T, 100 µL of 2,2’-azino-bis-(3-ethylbenzthiazoline-6-sulfonic acid) substrate solution (ABTS 2-Component Microwell Peroxidase Substrate Kit, SeraCare Life Sciences, Milford, MA, USA) was added and incubated for 30 min at 20–26 °C with shaking under a light shield. Absorbance was measured at 405 nm using a microplate spectrophotometer (Benchmark Plus, Bio-Rad Laboratories, Hercules, CA, USA), and the difference in absorbance between the rSFTSV-NP-coated and uncoated wells was calculated. The amount of anti-SFTSV NP antibody in the serum samples was calculated from the differences in absorbance of the rabbit anti-SFTSV IgG standard using a four-parameter logistic regression model (GraphPad Prism 6; GraphPad Software, La Jolla, CA, USA).

### Preparation of mouse anti-SFTSV NP antibody

The mouse anti-SFTSV NP antibody used in the neutralization assay was generated by DNA immunization. Five-week-old female ICR mice were purchased from KBT Oriental (Saga, Japan). The mice were injected with 50 µg of an expression plasmid, a pCI vector (Promega, Madison, WI, USA) encoding the NP gene of the SFTSV YG1 strain (Accession No. AB817995), using a Twin-Jector EZII jet injector (Japan Chemical Research, Ashiya, Japan). DNA injection was performed every two weeks, and the antiserum was separated from whole blood collected from the mice after the fourth immunization. The whole blood was collected from the heart without thoracotomy under inhalation anesthesia with isoflurane, and the mice were euthanized by total blood collection and cervical dislocation.

### Neutralization test

A 50% focus reduction neutralization test (FRNT_50_) was performed to analyze neutralizing antibody titers against SFTSV. Serum samples were diluted 1:5 and heat-treated at 56 °C for 30 min to inactivate the complement. The serum samples were further diluted 2-fold from 1:10 to 1:160, and equal volumes of the SFTSV A17 strain (accession Nos. LC536536, LC536546, and LC536556) adjusted to approximately 3,000 focus forming units/mL were added [30]. A serum-free dilution containing SFTSV was used as control. After incubation of the serum-virus mixture at 37 °C for 1 h, 100 µL of the incubated mixture was applied to Vero cell monolayers in a 24-well plate. After removal of the mixture and washing with PBS, the cells were overlaid with maintenance medium containing 1% methylcellulose and incubated in a 5% CO_2_ incubator at 37 °C. After 3 days, the cells were fixed with 4% paraformaldehyde for 1 h. After two washes with PBS, 200 µL of a 3,000-fold diluted mouse anti-SFTSV NP antibody was added to the cells and incubated for 1 h at 37 °C. After three washes with PBS, 200 µL of a 2,500-fold diluted goat anti-mouse IgG antibody (AlexaFluor488 anti-mouse IgG, Thermo Fisher Scientific) was added and incubated for 1 h at 37 °C. After three washes with PBS, the number of focal reactions in each diluted serum sample was counted using a fluorescence microscope (EVOS M7000 Imaging System; Thermo Fisher Scientific). The rate of reduction in the number of foci relative to that in the control at each serum dilution was calculated. If the rate of focus reduction was less than 50% at a 10-fold serum dilution or greater than 50% at a 160-fold serum dilution, the FRNT_50_ was defined as < 10 and > 160, respectively. If the FRNT_50_ was between 10 and 160, the FRNT_50_ was calculated using a four-parameter logistic regression model (GraphPad Prism 6). Neutralization was defined as positive if the FRNT_50_ was ≥ 10.

### RNA extraction and detection of SFTS viral RNA

Residual serum of 20 µL per feral horse was pooled from a maximum of 10 animals, regardless of the antibody test results. Viral RNA was extracted from each pooled 200 µL serum using a fully automated nucleic acid extraction system (MagLEAD system, Precision System Science, Chiba, Japan). The method for RNA extraction from ticks has been described in our previous report [28]. RNA was extracted from each individual tick.

One-Step PrimeScript III RT-qPCR Mix (TaKaRa Bio, Kusatsu, Japan) and PrimeTime qPCR assays (Integrated DNA Technologies, Coralville, IA, USA) with target gene-specific primers and probes were used to detect SFTSV and internal control genes of horses and ticks. The primers and probe set used for SFTSV, GAPDH and tick 18 S rDNA detection were adapted from previous reports using slightly modified primers and probe [31–33]. SFTSV forward primer; TGTCAGAGTGGTCCAGGATT, reverse primer; ACCTGTCTCCTTCAGCTTCT, probe; FAM-TGGAGTTTG/ZEN/GTGAGCAGCAGC-Iowa Black FQ. GAPDH forward primer; GCTGCCCAGAACATCATCC, reverse primer; GTCAGATCCACRACKGAYAC, probe; TEX-TCACTGGCATGGCCTTCCGT- Iowa Black RQ. Tick 18 S rDNA forward primer; AGCTAATACATGCAGTGAGC, reverse primer; TGATCGCATGGCCACGAG-3’, probe; Cy5-CGGGTGCTT/TAO/TTATTAGACC AAGAT-Iowa Black RQ. Sera from an SFTSV-infected cat and a tick engorged into the animal were used as positive controls.

### Statistical analysis

The chi-square test and Fisher’s exact test were used to compare viral infection rates between age groups and sexes, respectively. These analyses were performed using the GraphPad Prism 6 software. *p* < 0.05 was considered statistically significant.

## Results

To determine the cutoff ELISA value in horses, 293 serum samples from active Thoroughbred racehorses were tested using double-antigen ELISA. Almost all racehorses (99.7%, 292/293) showed low absorbance differences (mean, 0.0029; standard deviation (SD), 0.0038), and these 292 racehorses were considered a naive population that had never been exposed to SFTSV. In this study, the cutoff value was set at 0.0257, which was the mean of the absorbance difference for the naive population plus six times its SD. Only one racehorse with a high absorbance difference (0.776) in the ELISA was positive in the neutralization test (FRNT_50_: 51.6). Accordingly, this horse was excluded from the naive population.

**Fig. 1:**
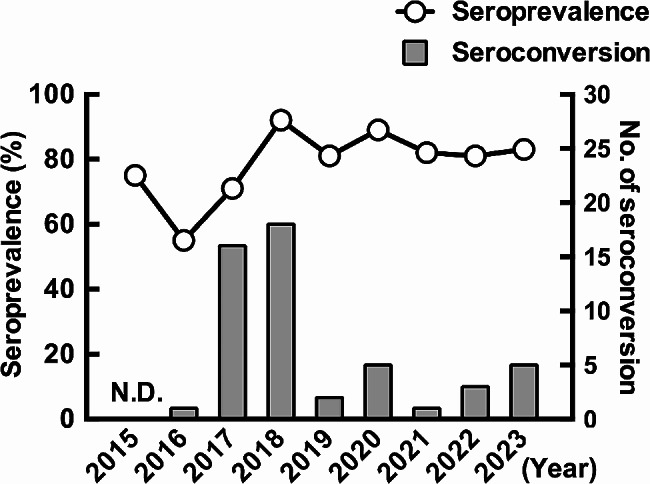
Seroprevalence and number of seroconversions to SFTS virus in feral horses at Cape Toi. The line graph shows seroprevalence, and the bar graph shows the number of seroconversions. The left *y*-axis shows seroprevalence, and the right *y*-axis shows the number of seroconversions. The number of seroconversions was not recorded for 2015 because no data were available for 2014

The seroprevalence of SFTSV in feral horses was analyzed using a double-antigen ELISA. The overall seroprevalence of feral horses from 2015 to 2023 was 78.5% (555/707) (Table 1). The seroprevalence was 75% (64/85) in 2015, decreased to 55% (44/80) in 2016, and increased to 71% (61/86) and 92% (76/83) from 2017 to 2018. After decreasing to 81% (68/84) in 2019, it increased to 89% (64/72) in 2020 and then decreased to 82% (55/67) in 2021 and was stable at 81% (56/69) in 2022 and 83% (67/81) in 2023. Seroprevalence differed significantly by age: 11% (8/71) were seropositive at less than one year of age, but 47% (33/71) at one year of age, 63% (40/64) at two years of age, 77% (39/51) at three years of age, and 96.7% (435/450) at four years of age or older were seropositive (*p* < 0.0001, chi-square test) (Table 1). The number of seroconversions from negative to positive varied from year to year, with 16 horses in 2017 and 18 in 2018, but less than five in other years (Fig. 1). The seroprevalence in male and female horses was 74.0% (245/331) and 82.4% (310/376), respectively (*p* < 0.01, Fisher’s exact test). In contrast, there was no difference in seroprevalence by sex in animals four years of age and older (male; 96.6% (197/204), female; 96.7% (238/246), *p* = 1.00, Fisher’s exact test). Feral horses less than one year of age represented 12.1% (40/331) and 8.2% (31/376) of male and female horses, respectively. The difference in the proportion of juveniles with low seroprevalence may have influenced the differences in seroprevalence according to sex.

**Table 1:** Seroprevalence of severe fever with thrombocytopenia syndrome virus in feral horses in Cape Toi, Japan

Year	Total	Male	Female	< 1-year-old	1-year-old	2-year-old	3-year-old	≥ 4-year-old
2015	75% (64/85)	72% (31/43)	79% (33/42)	30% (3/10)	38% (3/8)	40% (4/10)	83% (5/6)	96% (49/51)
2016	55% (44/80)	63% (22/35)	49% (22/45)	0% (0/10)	10% (1/10)	29% (2/7)	36% (4/11)	88% (37/42)
2017	71% (61/86)	67% (26/39)	75% (35/47)	10% (1/10)	56% (5/9)	75% (9/12)	60% (3/5)	86% (43/50)
2018	92% (76/83)	90% (35/39)	93% (41/44)	17% (1/6)	78% (7/9)	100% (10/10)	100% (9/9)	100% (49/49)
2019	81% (68/84)	78% (32/41)	84% (36/43)	11% (1/9)	22% (2/9)	88% (7/8)	100% (6/6)	100% (52/52)
2020	89% (64/72)	82% (28/34)	95% (36/38)	33% (1/3)	50% (3/6)	57% (4/7)	100% (6/6)	100% (50/50)
2021	82% (55/67)	70% (23/33)	94% (32/34)	0% (0/7)	67% (2/3)	33% (1/3)	50% (1/2)	98% (51/52)
2022	81% (56/69)	71% (22/31)	90% (34/38)	10% (1/10)	40% (2/5)	67% (2/3)	100% (2/2)	100% (49/49)
2023	83% (67/81)	72% (26/36)	91% (41/45)	0% (0/6)	67% (8/12)	25% (1/4)	75% (3/4)	100% (55/55)
Total	78.5% (555/707)	74.0% (245/331)	82.4% (310/376)	11% (8/71)	47% (33/71)	63% (40/64)	77% (39/51)	96.7% (435/450)

To ensure the reliability of the ELISA, neutralizing antibody titers against SFTSV were determined in serum samples from feral horses. In this study, 118 of 707 samples were used for neutralization testing. There were 37 serum samples with an FRNT_50_ less than 10, of which 34 were negative by ELISA (Table 2). There were 81 serum samples with FRNT_50_ greater than 10, of which 71 tested positive by ELISA. All 41 serum samples with an FRNT_50_ greater than 160 were positive by ELISA. The concordance rate between ELISA and neutralization test results was 88.9% (105/118).

**Table 2:** Correlation between neutralization assay and ELISA results using horse serum

FRNT_50_^†^	Absorbance difference		Antibody concentration (ng/mL)		
	< 0.0257	≥ 0.0257	< 1096^‡^	1096–3641^‡^	≥ 3641^‡^
	Negative	Positive	Negative	Weakly positive	Positive
< 10	34	3	34	3	0
10–40	7	4	8	3	0
40–160	3	26	2	4	23
> 160	0	41	0	0	41
Total	44	74	44	10	64

^†^ FRNT_50_: 50% focus reduction neutralization test

^‡^ These cutoff values were calculated by Kaneko et al. [19]

As many feral horses have anti-SFTSV antibodies, we suspected that some were viremic. Therefore, we attempted to detect SFTS viral RNA in all serum samples using real-time reverse transcription PCR; however, it was not detected.

Next, 516 ticks collected from Cape Toi between November 2021 and November 2022 were tested for SFTS viral RNA. Of the 516 ticks collected. 301 were *Haemaphysalis longicornis* (230 nymphs and 71 adults), 138 were *H. formosensis* (79 nymphs and 59 adults), 70 were *H. flava* (62 nymphs and eight adults), four were *H. hystricis* (four adults), and three were *Amblyomma testudinarium* (two nymphs and one adult) [28]. However, no SFTS viral RNA was detected in the ticks.

## Discussion

This study encompassed a nine-year serological survey for SFTSV in feral horses in Miyazaki Prefecture, Japan, an SFTS-endemic area. Annual seroprevalence ranged from 55 to 92%, with the highest seroprevalence in 2018 and the lowest in 2016. Seroprevalence was considerably influenced by age, with only 11% of horses less than one year of age being seropositive, whereas 96.7% of horses four years of age and older were seropositive. The number of seroconversions was inconsistent annually, with higher numbers occurring in 2017 and 2018. Despite its high seroprevalence, SFTS viral RNA has not been detected in feral horses or vegetation ticks in their habitats.

A double-antigen ELISA using rSFTSV-NP was used in this study [29]. This method does not require the use of HRP-labeled secondary antibodies for serum samples from different species. Thus, serological tests for multiple species (e.g., dogs and cats) can be performed simultaneously, and wildlife for which secondary antibodies are difficult to prepare can be tested. We have previously reported an antibody test against SFTSV in humans, dogs, cats, Japanese badgers, and Japanese raccoons using this method [13, 19, 34]. However, the correlation between the values measured by ELISA and neutralizing activity against SFTSV has not been investigated. This study showed that the concordance rate between the ELISA and neutralization test results was 88.9%. A possible reason for the partial disagreement may be that the neutralization target was directed against the Gn/Gc glycoproteins of SFTSV, and the ELISA was directed against the NP [35]. Based on these results, this method may be a suitable and reliable tool for serological surveys of SFTSV in animals.

To perform ELISA using horse serum samples, a cutoff value had to be established, and a certain number of horse serum samples that had never been exposed to SFTSV was required. Approximately 98% of Thoroughbred racehorses in Japan are raised in Hokkaido [36], an area where no SFTS patients have been confirmed, and they are carefully managed by humans, indicating that they are less exposed to ticks. Therefore, we believe that they could serve as naive controls that had never been exposed to SFTSV. As expected, 99.7% (292/293) of the racehorses were considered naive, with a mean absorbance difference of 0.0029 and an SD of 0.0038. Because the mean and SD of the absorbance differences were quite low, the mean plus six times the SD was used as the cutoff value (0.0257) in this study. We have previously calculated cutoff values in the same ELISA method for wildlife (Japanese badger and Japanese raccoon dog) for which naive population serum was not available, using the change-point analysis method [19, 37]. If the cutoff values calculated in a previous study (antibody concentrations of ≥ 3641 ng/mL as positive, 1096–3641 ng/mL as weakly positive, and < 1096 ng/mL as negative) were used in this study, the seroprevalence of feral horses would be 79.1%, which is nearly the same as the seroprevalence in this study (Table 2). This result indicates that the cutoff values calculated by Kaneko et al. can be applied as cutoff values for double-antigen ELISA in any wild or companion animal [19].

In this study, 78.5% of the feral horses in Miyazaki had a history of SFTSV infection, and 55% (41/74) had high neutralizing antibody titers to SFTSV (FRNT_50_; >160). On the other hand, there was almost no history of infection in active Thoroughbreds. To the best of our knowledge, there are few reports on SFTSV infection in horses. Lee et al. [25] reported that approximately 20% of 70 horses on farms in South Korea, were antibody positive by ELISA and neutralization testing. Han et al. [26] reported that of the 887 horses in the slaughterhouse and equestrian centers in South Korea, 51% were antibody positive and 1.3% were positive for SFTSV RNA. These results indicate that horses can be infected with SFTSV and develop a short period of viremia. On the other hand, no horses have been reported to develop severe symptoms, thus, unlike cats and dogs [12–14], horses are not considered to be a source of direct non-tick-mediated transmission of the virus. While active Thoroughbreds are at little risk of being a source of SFTSV infection, the possibility that ticks engorged to horses can be a source of infection to humans cannot be ruled out in riding horses, food horses, and wild horses.

In Japan, the Kyushu-Yamaguchi area is most endemic for SFTS, and several serological studies have been conducted on wildlife. In particular, sika deer (*Cervus nippon*) and wild boar (*Sus scrofa*) have been designated by the Japanese government as control animals, and serum samples are relatively easy to obtain compared to those from other wildlife. Surveys conducted in Yamaguchi, Kagoshima, and Oita prefectures reported that 64.6% (*n* = 789, 2010–2020), 34.7% (*n* = 107, 2014–2018), and 54.8% (*n* = 115, 2011 and 2014) of sika deer were seropositive, respectively [22, 38, 39]. Surveys conducted in Yamaguchi, Kagoshima, Oita, Nagasaki, and Miyazaki prefectures reported that 38.5% (*n* = 517, 2010–2020), 53.9% (*n* = 102, 2014–2018), 12% (*n* = 65, 2011 and 2014), 18.9% (*n* = 190, 2006–2012), and 41.9% (*n* = 105, 2009) of wild boar were seropositive, respectively [22, 38–41]. In other wildlife, 68% (*n* = 63) of Japanese badgers (*Meles anakuma*) and 23% (*n* = 53) of Japanese raccoons (*Nyctereutes procyonoides viverrinus*) had antibodies against SFTSV in Miyazaki Prefecture between 2019 and 2021 [19]. Although not in the Kyushu-Yamaguchi area, a large-scale survey of raccoons (*Procyon lotor*) was conducted in Wakayama Prefecture over 12 years, with a mean seroprevalence of 39.8% (*n* = 2299) and no years exceeding 60% [23]. Compared with other wildlife, the seroprevalence of SFTSV tends to be higher in feral horses and sika deer. It is possible that these animals are more susceptible to SFTSV infection; however, they are not multiparous, and their long lifespans may have contributed to their high seroprevalence. Wild boars, raccoons, and badgers were multiparous. Deer and horses consume only one litter per year. A typical wildlife seroepidemiological survey cannot identify individuals or obtain information such as age. Misaki horses were individually identified and their ages were determined. The age of feral horses contributed remarkably to the seroprevalence of SFTSV, with seroprevalence reaching nearly 100% in horses four years of age and older compared to 11% in horses less than one year of age. Thus, the proportion of young animals can have a significant impact on the seroprevalence of SFTSV in wildlife. Wildlife with a higher frequency of generational change may have a lower seroprevalence of SFTSV in SFTS-endemic areas.

Based on the results of ELISA and neutralization tests, there is no doubt that feral horses in Miyazaki have been exposed to SFTSV. However, despite its high seroprevalence, SFTS viral RNA has not been detected. Blood samples were collected from the feral horses in September of each year. In Japan, SFTS tends to be most prevalent in May and least prevalent from November to January [27]. Therefore, the prevalence of SFTSV in ticks should not have decreased extensively during September. A possible explanation for this is that the seroprevalence in feral horses was too high, limiting the number of animals that could become viremic. Tatemoto et al. also reported high seroprevalence in wildlife but reported that SFTS viral RNA was detected in only 0.4% (*n* = 229) of serum samples collected from sika deer and 0% (*n* = 116) from wild boars [22]. In contrast, SFTS viral RNA was detected in 7.6% (*n* = 106) of wild boars in Miyazaki, but the seroprevalence was only 41.9% [41]. Higher seroprevalence of SFTSV does not appear to increase the detection of SFTS viral RNA in wildlife. When seroprevalence is extremely high, the endemicity of SFTSV in the population may be limited owing to the acquisition of herd immunity. When seroprevalence in the population decreases owing to generational changes, SFTSV could once more become endemic in the population.

In the present study, there was a marked difference in seroprevalence by sex among feral horses. However, because there was no difference in seroprevalence among animals four years of age and older, the study concluded that the difference was due only to the higher proportion of juveniles among male horses. A total of 331 males and 376 females were sampled, with 40 males (12.1%) and 31 females (8.2%) sampled from horses less than one year of age. However, it is unclear why male Misaki horses were born in higher numbers and had shorter lives.

Vegetation ticks were collected from Cape Toi at two-month intervals between November 2021 and November 2022; however, no SFTS viral RNA was detected. The fact that sampling was conducted only in a limited area of Cape Toi may also have affected the negative results. Further research is required to determine the prevalence of SFTSV in ticks in Cape Toi.

## Conclusions

This study demonstrated that horses can be infected with SFTSV and that a high percentage of feral horses in SFTS-endemic areas are exposed to SFTSV. Because no SFTS viral RNA was detected, we believe that horses are not natural hosts of SFTSV. Research on Misaki horses, which are wild but have been individually identified, will help us understand the spread of SFTSV in wildlife. Continued research is important to understand the transmission cycle of SFTSV in nature and to identify unidentified natural host species. Surveillance of SFTSV infections in wildlife is particularly important for predicting SFTS outbreaks in humans.

## Data Availability

The data that support the findings of this study are available from the corresponding author upon reasonable request.

## Abbreviations

ELISA: Enzyme-linked immunosorbent assay
FRNT_50_: 50% focus reduction neutralization test
HRP: Horseradish peroxidase
NP: Nucleocapsid protein
rSFTSV-NP: Recombinant severe fever with thrombocytopenia syndrome virus nucleocapsid protein
PBS: Phosphate-buffered saline
SD: Standard deviation
SFTS: Severe fever with thrombocytopenia syndrome
SFTSV: Severe fever with thrombocytopenia syndrome virus
TBS-T: Tris-buffered saline with Tween 20
